# Quantifying the feeding behavior and trophic impact of a widespread oceanic ctenophore

**DOI:** 10.1038/s41598-023-27955-z

**Published:** 2023-02-09

**Authors:** Betsy Potter, Marco Corrales-Ugalde, James P. Townsend, Sean P. Colin, Kelly R. Sutherland, John H. Costello, Richard Collins, Brad J. Gemmell

**Affiliations:** 1 Integrative Biology, University of South Florida, Tampa, FL USA; 2 Atmospheric and Oceanic Sciences, Princeton University, Princeton, NJ USA; 3 Providence College, Biology, Providence, RI USA; 4 Marine Biology, Roger Williams University, Bristol, RI USA; 5 Oregon Institute of Marine Biology, University of Oregon, Eugene, OR USA; 6 Florida Museum of Natural History, Gainsville, FL USA

**Keywords:** Ocean sciences, Behavioural ecology

## Abstract

Oceanic ctenophores are widespread predators on pelagic zooplankton. While data on coastal ctenophores often show strong top-down predatory impacts in their ecosystems, differing morphologies, prey capture mechanisms and behaviors of oceanic species preclude the use of coastal data to draw conclusion on oceanic species. We used high-resolution imaging methods both in situ and in the laboratory to quantify interactions of *Ocyropsis* spp. with natural copepod prey. We confirmed that *Ocyropsis* spp. uses muscular lobe contraction and a prehensile mouth to capture prey, which is unique amongst ctenophores. This feeding mechanism results in high overall capture success whether encountering single or multiple prey between the lobes (71 and 81% respectively). However, multiple prey require several attempts for successful capture whereas single prey are often captured on the first attempt. Digestion of adult copepods takes 44 min at 25 °C and does not vary with ctenophore size. At high natural densities, we estimate that *Ocyropsis* spp. consume up to 40% of the daily copepod standing stock. This suggests that, when numerous, *Ocyropsis* spp. can exert strong top-down control on oceanic copepod populations. At more common densities, these animals consume only a small proportion of the daily copepod standing stock. However, compared to data from pelagic fishes and oceanic medusae, *Ocyropsis* spp. appears to be the dominant copepod predator in this habitat.

## Introduction

Carnivorous gelatinous zooplankton such as ctenophores and cnidarians, are ubiquitous across oceanic marine ecosystems^1^. However, key variables that define their trophic dynamics, such as predatory efficiency and ingestion rates have been poorly studied compared to other taxonomic groups such as crustaceans and fishes. Most of what is known about the feeding capabilities and trophic impacts of ctenophores come from studies on coastal species, due to ease of access, robustness in laboratory settings^2,3^, and predictable seasonal abundances^4–6^. In contrast, oceanic ctenophores are understudied, since traditional sampling methods often destroy these delicate, soft-bodied organisms and the highly sensitive nature of these animals makes it difficult to keep them alive in captivity. Thus, in situ observations and imaging methods are often needed to gather relevant biological data^7^. Recent advances in imaging technology allow undisturbed observations with high spatial and temporal resolution of the morphology, behavior and species interactions of pelagic oceanic ctenophores, in order to address questions regarding their feeding ecology and trophic impacts.

Populations of oceanic gelatinous zooplankton are often patchy, and densities can vary up to two orders of magnitude over short distances^8^. In the Atlantic, ctenophores have been found at densities of 0 to over 1000 individuals per 1000 m^3^^8,9^. Population patchiness is often attributed to variations in large scale physical processes such as oceanic currents, latitudinal differences, and large-scale temperature shifts mediated by climate change, as well as small scale processes such as eddies, and turbulent diffusion^8,10^. When abundant, ctenophores serve as important grazers on zooplankton^11–13^ and through their widespread distributions, they can also contribute to the global carbon pump through deposition of oceanic carbon to the seafloor through events known as ‘jelly falls’^14^. Considering the ubiquity of many ctenophore taxa^8^, it is important to gain a better understanding of the trophic role of oceanic ctenophores in this ecosystem.

One of the most commonly encountered oceanic ctenophores belong to the family Ocyropsidae. This group is unique amongst ctenophores because they lack tentillae and colloblasts, the primary prey capture surfaces of other species^15^. Instead, *Ocyropsis* spp. use large muscular lobes and a rapidly moving prehensile mouth to capture fast-swimming, evasive prey such as copepods^15^. This active feeding mechanism causes a brief interruption in foraging, but qualitative observations in situ suggest that it allows them to capture larger and more active prey than other lobate species^9^, which may result in higher overall biomass ingestion. Prey capture involves hydrodynamic detection of prey movements or direct contact with an inner lobe and an instantaneous contraction of the oral lobe at the contact point where the prehensile mouth reaches to collect the prey for ingestion^15^. This feeding interaction is considered more direct than those observed in ctenophore species that capture prey using tentacles and/or colloblasts^15^.

When feeding, *Ocyropsis* spp*.* typically propel themselves horizontally through the water using their ctene rows at consistent speeds around 14 mm s^−1^, but they are also capable of a short bursts of rapid swimming by flapping their oral lobes in a manner similar to the swimming of a clam^15^. *Ocyropsis* spp. escape swimming can reach speeds of 125 mm s^−1^^16^ and can do an average of 1 to 6 continuous flaps^15^. This behavior is thought to be a means to avoid predators or quickly reposition in a new patch of water that may contain more prey. The unique prey capture mechanism of *Ocyropsis* spp*.* must be able to sustain it in waters with low prey densities. Oceanic copepod densities off the coast of southeastern Florida range from 150 to 1700 individuals m^3^^17–19^, while coastal prey densities are often much higher reaching > 3600 individuals m^3^^20,21^. *Ocyropsis* spp. themselves have patchy distributions and have been observed at densities up to 1 individuals m^3^^9,15^. Such high densities of macroscopic zooplankton predators in an oceanic environment have the potential to exert a strong top-down impact on smaller zooplankton populations such as copepods.

The purpose of this study was to quantify predator–prey interactions of *Ocyropsis* spp. Using high resolution videography and photography in laboratory and in situ settings, we quantified the prey capture efficiency, kinematics of predator–prey interactions, prey handling, gut fullness, and digestion time of *Ocyropsis* spp. These data were then used to approximate the maximum trophic impact of *Ocyropsis* spp. in oceanic waters of eastern Florida.

## Methods

Collection and in situ imaging of *Ocyropsis* spp. were made via blue-water (daytime) and black-water (nighttime) SCUBA diving from a small boat along the western edge of the Gulf Stream, 5 to 8 km off the coast of West Palm Beach, Florida (26° 43′ 93″ N, 79° 59′ 15″ W). On all dives, *Ocyropsis* spp. were found in surface waters, and all imaging and collection took place within the upper 15 m of the water column. Animals were hand-collected by SCUBA divers using 1 L jars and transported back to the laboratory for observations. All images used in the manuscript were taken by the authors.

### Predator–prey interactions

Ctenophores (n = 25) from the field were held at a constant temperature of 25 °C and filmed within 12 h of collection. Copepods were collected using a 30 cm diameter, 150 µm mesh plankton net at the surface (depth of 1 m). Copepods were roughly sorted by size through sieves (200 and 500 µm mesh) prior to experiments. Individual ctenophores were gently placed into a 4 L filming vessel with temperature-matched sea water collected offshore. A Sony AX100 camera with brightfield illumination recording in 4 K resolution was used to record observations. After a 10-min acclimation time, video recording commenced and copepods were added to the tank using a wide-bore pipette. All video sequences were converted to image stacks and analyzed using ImageJ to calculate the average speed, maximum speed, and total displacement of the copepods and ctenophore mouth, handling time, capture success per attempt, overall success rate and the number of predation attempts made by the ctenophore.

**Figure 1:**
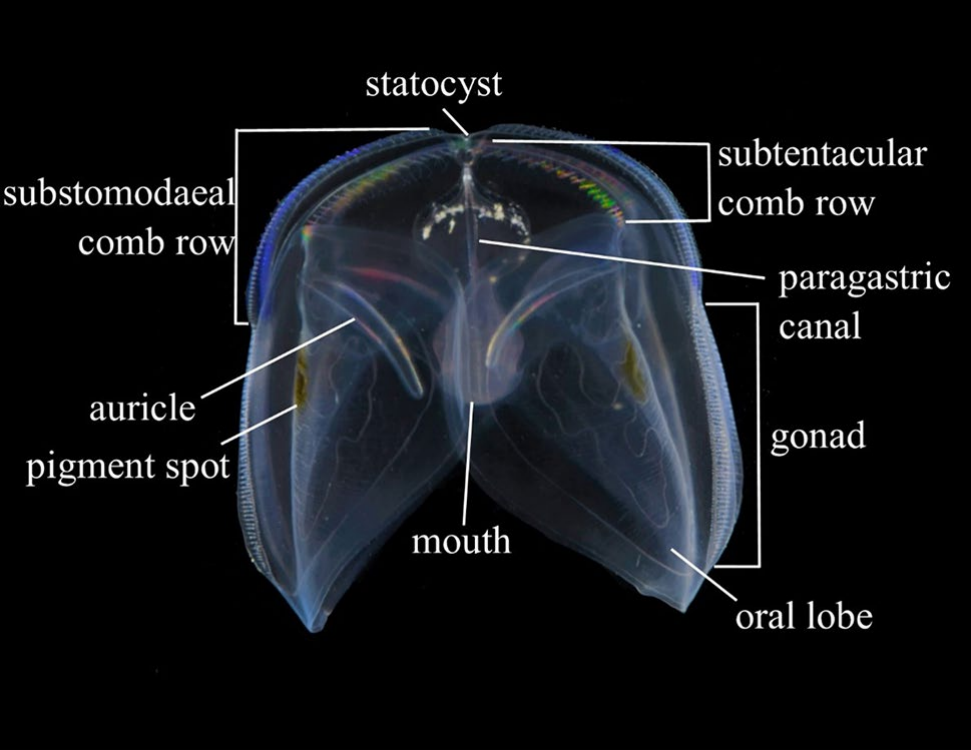
Anatomically labeled image of *Ocyropsis maculata*. With the exception of the pigment spots, all features noted here are found and arranged in the same manner in all known species of *Ocyropsidae*.

An encounter was initiated when a ctenophore physically responded to hydrodynamic (or physical) stimulus from a copepod. Two types of encounters were observed: (1) a single prey item present between the lobes (single copepod encounter) and (2) multiple copepods present between the lobes (multiple copepod encounter). For single copepod encounters, movement of both the copepod and the ctenophore mouth were tracked. Multiple copepods made individual tracking too difficult and therefore only the mouth was tracked. Handling time was defined as the time from the initial ctenophore response to prey to either prey ingestion or to prey escape and a return of the mouth to the initial resting position (Fig. 1). The mouth would often make multiple attempts to capture prey if it failed on the first attempt, thus capture success per attempt as well as the overall success rate were record.

Because data violated assumptions of normality, we used a non-parametric Spearman’s Rank correlation tests to determine whether there were significant relationships between the number of attempts made per interaction and capture success rate, handling time, total copepod displacement, and total mouth displacement. A linear regression was performed to assess the relationship between handling time and total mouth displacement data. Welch’s two sample *t* tests were used to compare handling times, total mouth displacement, and average mouth speed between the two types of prey encounters (single or multiple). Mann–Whitney *U* tests were used to compare data for mean capture success rates and number of attempts between single and multiple copepod encounters. An alpha value of 0.05 was used for all statistical tests, and all data was checked to ensure test-specific assumptions were met for each statistical test performed.

### Gut content analysis

Still images (36.3 Megapixel) and high-resolution 4 K video frames showing a clear view of the gut were used to obtain in situ gut contents and ctenophore size. Number of prey items inside the gut, average prey item length, total prey biomass, and percent gut fullness were measured. Biomass of individual prey items was calculated by assuming a cylindrical body shape and extracting each individual prey’s volume. Then, assuming each prey item had the approximate density of seawater—a conservative estimate given that zooplankton are slightly negatively buoyant—biomass was calculated. Mann–Whitney *U* tests were used to compare mean number of prey, average prey length, total prey biomass, and gut fullness from daytime and nighttime gut content analyses. An Analysis of Covariance (ANCOVA) was used to determine whether the length of an individual ctenophore acted as a covariant with time of day to affect percent gut fullness. Linear regressions were used to compare relationships between ctenophore body length and total prey biomass, average prey length, percent gut fullness, and number of prey per gut.

### Digestion rates

Individuals of *Ocyropsis* spp. were held unfed for 12 h at a temperature of 25 °C. Ctenophores were offered a mixed assemblage of copepods from the genera *Acartia, Oncaea,* and *Microsetella*. Digestion time observations were made using a Motic SMZ-171 stereo microscope and began immediately following the first observation of prey ingestion. Observations continued every two minutes until digestion was complete. High resolution image series of digestion were also made for several individual ctenophores using a Nikon 750 DLSR camera coupled to the stereo microscope. Complete digestion was defined as the time at which the only visible remains of the copepod prey were chitinous structures.

### Zooplankton assemblage quantification

Plankton tows were performed using a 30 cm diameter, 150 µm mesh plankton net. The net was towed just below the surface (approximately 1 m) for 2–3 min. Real time speed was recorded with onboard GPS and time for each tow (to the nearest second) was also recorded. Samples were collected during both day and night. Immediately after collection, the samples were fixed in a 10% ethanol/seawater solution as it provides good zooplankton preservation. Sub samples were extracted from well mixed sample jars using a Hensen-Stempel Pipette and quantified using a Motic SMZ-171 stereo microscope at 4-5X magnification.

### Predation rate and trophic impacts

Predation rates were calculated using high and low-end estimates of *Ocyropsis* spp. density from Harbison, et al.^9^ and the following equation from Pagès, et al.^22^:

1 $$I=M\times \left(\frac{C}{{M}_{e}}\right)\times \left(\frac{1}{D}\right)\times 24,$$

where M is the density of ctenophores (ind m^−3^), C is the total number of prey in the gut, M_e_ is the number of ctenophores measured, D is digestion time in hours, and I is the number of copepods ingested (ind m^−3^ d^−1^). Potential consumption rate was calculated by dividing the predation rate of one ctenophore by the low-end copepod standing stock value from the daytime net tow. An estimate from Kremer, et al.^23^ of carbon content of tropical copepod species (2.5 µg/copepod) was multiplied by the total biomass of copepods consumed by one ctenophore in one day to calculate carbon ingested per *Ocyropsis* spp. per day.

## Results

### Description of the interaction

**Figure 2:**
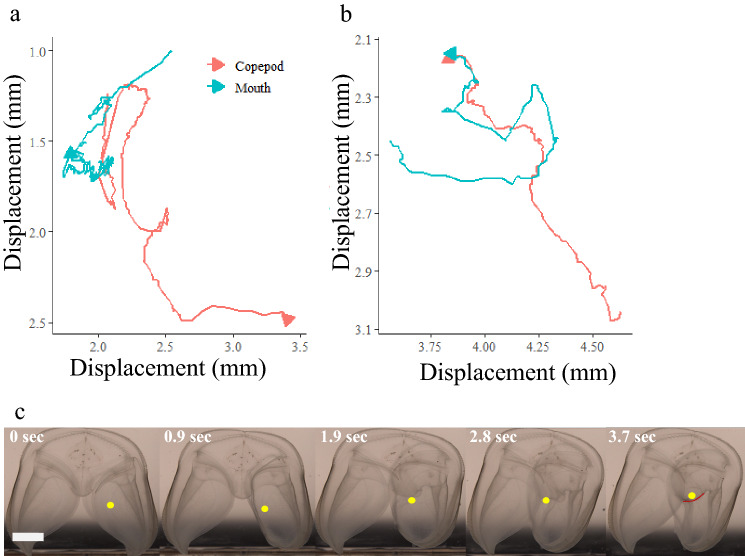
Example of 2-dimensional displacements of both copepod and ctenophore mouth for two predation attempts. Arrows indicate ending points. (**a**) copepod escape, encounter lasted 6.7 s, (**b**) copepod capture, encounter lasted 3.7 s. Axes have been adjusted to better display details of the encounter. (**c**) sequential images displaying the encounter in panel (**b**). Yellow dots show copepod location, red curve in the final photo outlines the edge of mouth to show that the copepod has been consumed. Scale bar in first image represents 5 mm.

Prior to sensing copepod prey, ctenophores were typically observed hovering or slowly cruising with aboral end up, lobes outstretched and auricle cilia beating in a relaxed position (Fig. 1). Ctenophores responded to hydrodynamic signals of a swimming copepod between the lobes by folding or contracting the lobes around the position of the copepod. This action either trapped the copepod so that it could no longer move or cut off possible escape paths, thereby isolating the copepod to a small area of the lobe(s), while the dexterous mouth instantly began searching for the prey (Fig. 2). As the mouth continued to seek out the copepod, the point at which the lobe folded or contracted often moved closer to the mouth. Some encounters involved a direct transfer of the trapped copepod into the mouth. For encounters where the lobe contraction only isolated the copepod to a smaller space within the lobe(s), the mouth often moved in that direction to the extent of its reach and opened, stretched, and changed shape while pursuing the copepod until the copepod was consumed or eventually escaped. After successful ingestion of a copepod or an unsuccessful “chasing” event, whereby the mouth actively pursues the copepod trapped between the lobes, the ctenophore would relax any contraction in the lobes and the mouth would slowly return to the initial resting position.

### Single copepod encounters

A total of 35 encounters between a single ctenophore and a single copepod prey were assessed for handling time and capture success. Average handling time for natural copepod prey was 6.34 s (S.D. 4.49), and median handling time was 4.56 s (Table 1). The average capture success rate on the first attempt was 60.9% (S.D. 45.2) while the overall success rate (success after all attempts combined) was 71.4% (S.D. 45.8) (Table 1).

**Table 1:** Mean (S.D.) and median values for parameters measured in both single and multiple copepod encounters.

		N encounters	Handling time (s)	Attempts	Overall success rate (%)	1st attempt success rate (%)	Total mouth displacement (mm)	Average mouth speed (mm^−1^)	Maximum mouth speed (mm^−1^)
Single copepod encounters	Mean (S.D.)	35	6.34 (4.49)	1.34 (0.68)	71.4 (45.8)	60.9 (45.2)	36.1 (22.5)	5.83 (1.68)	38.8 (40.0)
	Median		4.56	1			29.8	5.98	31.94
Multiple copepod encounters	Mean (S.D.)	21	16.4 (9.37)	3.24 (1.58)	80.9 (40.2)	0.00	111.0 (71.8)	5.58 (1.94)	36.3 (21.7)
	Median		13.5	3			94.2	6.05	32.96

**Figure 3:**
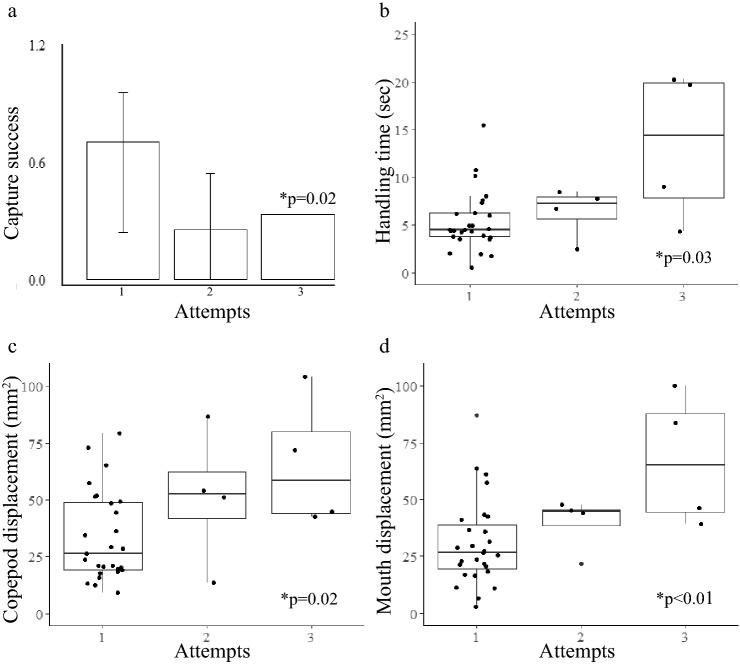
Relationship between number of attempts made in one encounter and (**a**) capture success rate (only one encounter contained three attempts) (*p* = 0.02, rho = − 0.38), (**b**) handling time (*p* = 0.03, rho = 0.37), (**c**) total copepod displacement (*p* = 0.02, rho = 0.38), and (**d**) total mouth displacement (*p* < 0.01, rho = 0.46). Thick horizontal lines within each box show median values, vertical lines on either side of each box show standard error, dots represent individual data points.

The number of attempts made during an encounter was negatively correlated with capture success rate (Spearman, *p* = 0.02, n = 35). Number of attempts was positively correlated with the following parameters: handling time (Spearman, *p* = 0.03, n = 35), total copepod displacement (Spearman, *p* = 0.02, n = 35), and total mouth displacement (Spearman, *p* < 0.01, n = 35) (Fig. 3). The maximum number of attempts made in any of the single copepod encounters was three attempts, mean number of attempts was 1.34 (S.D. 0.68), and median number of attempts was 1 (Fig. 3, Table 1). These correlations showed that if the first attempt was not successful, chance of capture decreased by half and capture required more time.

**Figure 4:**
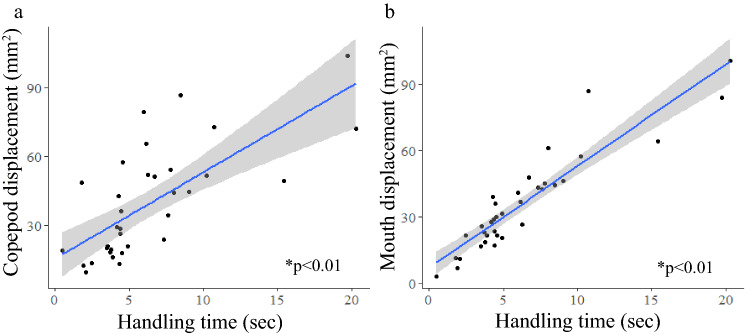
Linear regressions of single copepod encounter data showing significant relationships between handling time and (**a**) total copepod displacement (*p* < 0.01, r^2^ = 0.49) and (**b**) total mouth displacement (*p* < 0.01, r^2^ = 0.84). Statistical calculations were performed using log transformations, but data are shown with no transformations. Grey shaded region shows 95% confidence interval.

There was a significant positive linear relationship between handling time and total copepod displacement (linear regression, *p* < 0.01, r^2^ = 0.49, n = 35) and handling time and total mouth displacement (linear regression, *p* < 0.01, r^2^ = 0.84, n = 35) (Fig. 4). Longer encounters resulted in more movement of both the predator and prey, as well as led to more attempts made by the mouth (Figs. 3b, 4). Thus, there was a significant positive linear relationship between total mouth displacement and total copepod displacement (linear regression, *p* < 0.01, *r*^*2*^ = 0.49, n = 35). We found significant differences between the handling times for single copepods and the time for handling multiple copepod encounters (*t* test, *p* < 0.01, n = 56). We also found significant differences between total mouth displacement during single versus multiple copepod encounters (*t* test, *p* < 0.01, *n* = 56) (Table 1). Handling time and displacement of the mouth were significantly lower in single copepod encounters than in multiple copepod encounters (*t* test, *p* < 0.01, n = 56; *t* test, *p* < 0.01, n = 56) (Table 1). Ctenophores did not capture any prey on the first attempt in which multiple copepods were encountered, so the average capture success rate of single copepod encounters, 60.9% (S.D. 45.2), showed that fewer attempts were needed when only one prey item was present (Mann–Whitney: *p* < 0.01, n = 56).

### Multiple copepod encounters

A total of 21 encounters were analyzed where multiple copepods were present between the lobes during a predation event. Mean handling time was 16.4 s (S.D. 9.37), and median handling time was 13.5 s (Table 1). Here, the capture success rate on the first attempt was 0%, while the overall success rate was 80.9% (S.D. 40.2) during a predation event (Table 1). Despite no encounters resulting in capture on the first attempt, having multiple copepods present at one time increased the likelihood of at least one capture overall by 9.5%. The average mouth speed was 5.58 mm s^−1^ (S.D. 1.94) (median: 6.05 mm s^−1^), which was not significantly different from average mouth speed of single copepod encounters (5.83 mm s^−1^ (S.D. 1.68)) (median: 5.98 mm s^−1^) (*t* test, *p* = 0.627, n = 56) (Table 1). Maximum mouth speed was also not significantly different between the two types of encounters (*t* test, *p* = 0.76, n = 56).

**Figure 5:**
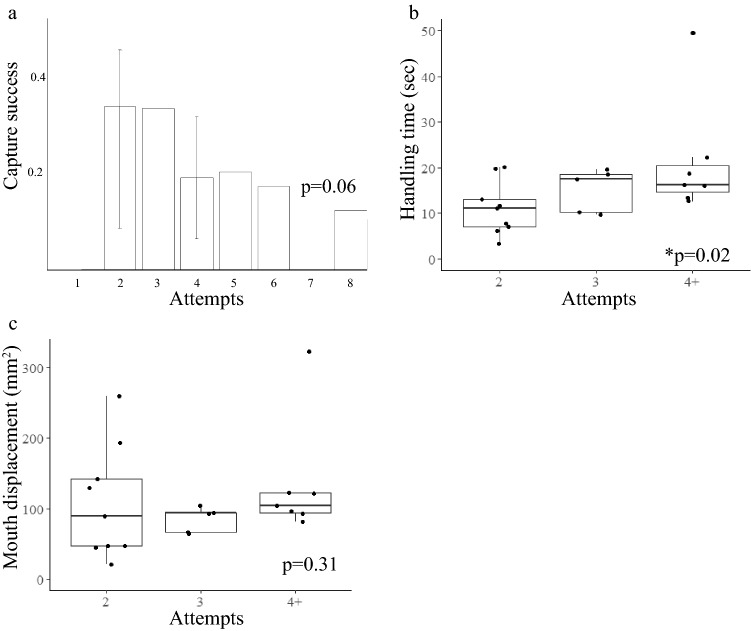
Interactions with multiple copepods and the relationship between number of attempts taken in one encounter and (**a**) capture success rate (*p* = 0.06, rho = − 0.42); values on the x-axis without error bars represent a number of attempts that was seen in only one encounter, (**b**) handling time (*p* = 0.02, rho = 0.50), and (**c**) total mouth displacement (*p* = 0.31, rho = 0.23). Thick horizontal lines within each box show median values, vertical lines on either side of each box show standard error, dots represent individual data points.

**Figure 6:**
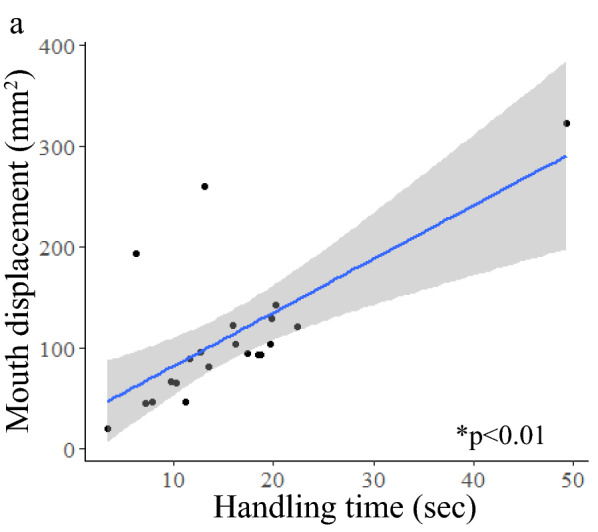
Linear regression of multiple copepod encounter data showing a significant relationship between handling time and total mouth displacement (*p* < 0.01, r^2^ = 0.47). Grey shaded region shows 95% confidence interval. Statistical calculations were performed using a log transformation. Data in figure are shown without transformations.

The mean number of predation attempts made by *Ocyropsis* when multiple prey items were present between the lobes (3.24 attempts (S.D. 1.58)) was greater than when only a single individual was present (1.34 attempts (S.D. 0.68)) (Mann–Whitney, *p* < 0.01, n = 56). There was no significant correlation between number of attempts made and capture success rate (Spearman, *p* = 0.06, n = 21) (Fig. 5a). Unlike single copepod encounter events, the number of attempts made within one encounter did not correlate with displacement of the mouth (Spearman, *p* = 0.31, n = 21) (Fig. 5c). Some encounters involved many attempts that were small movements targeted in one concentrated area, while others involved sequential attempts in which the mouth moved from one side of the lobes to the other. There was a significant positive correlation between attempts made and handling time (Spearman, *p* = 0.02, n = 21) (Fig. 5b). No encounters resulted in prey capture on the first attempt. Additionally, there was a significant positive linear relationship between total mouth displacement and handling time (linear regression, *p* < 0.01, n = 21) (Fig. 6).

### In situ gut content and plankton net community assessment

**Figure 7:**
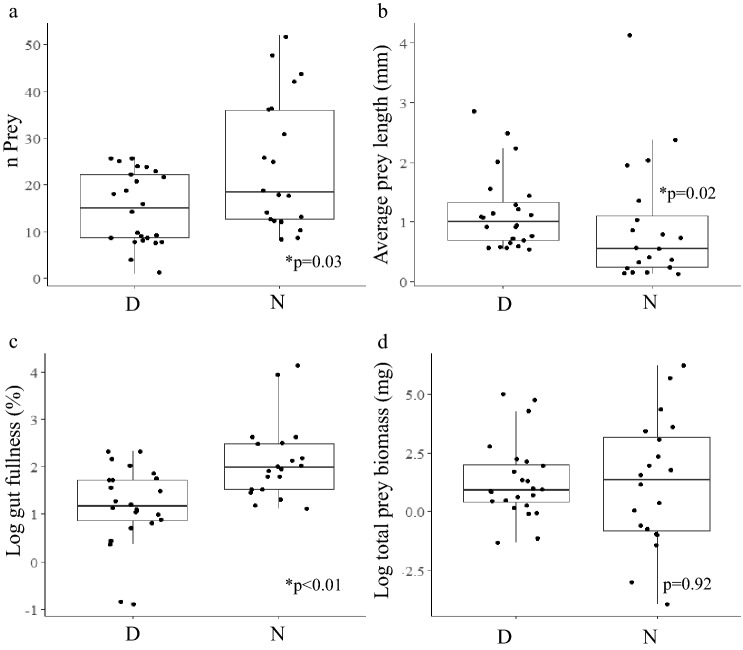
Boxplots showing differences between guts measured during day (D) and night (N) and (**a**) number of prey per gut (*p* = 0.03, U = 148), (**b**) average prey length (*p* = 0.02, U = 334) (**c**) percent gut fullness, log transformed (*p* < 0.01, U = 93), and (**d**) total prey biomass per gut, log transformed (*p* = 0.92, U = 245). Thick horizontal lines within each box show median values, vertical lines on either side of each box show standard error, dots represent outliers.

Mann–Whitney *U* tests were used to compare in situ gut content parameters due to non-normal distribution. Because prey within guts were in different states of digestion, it was not possible to identify their genera or species. Nighttime sampling showed significantly more prey items present in guts (Mann–Whitney, *p* = 0.03, n = 44). Prey were significantly smaller (Mann–Whitney, *p* = 0.02, n = 44) and ctenophores had significantly higher gut fullness (Mann–Whitney, *p* < 0.01, n = 44) at night (Fig. 7a–c). While the aforementioned parameters differed, there was no significant difference of biomass per gut between day and night (Mann–Whitney, *p* = 0.92, n = 44) (Fig. 7d).

**Figure 8:**
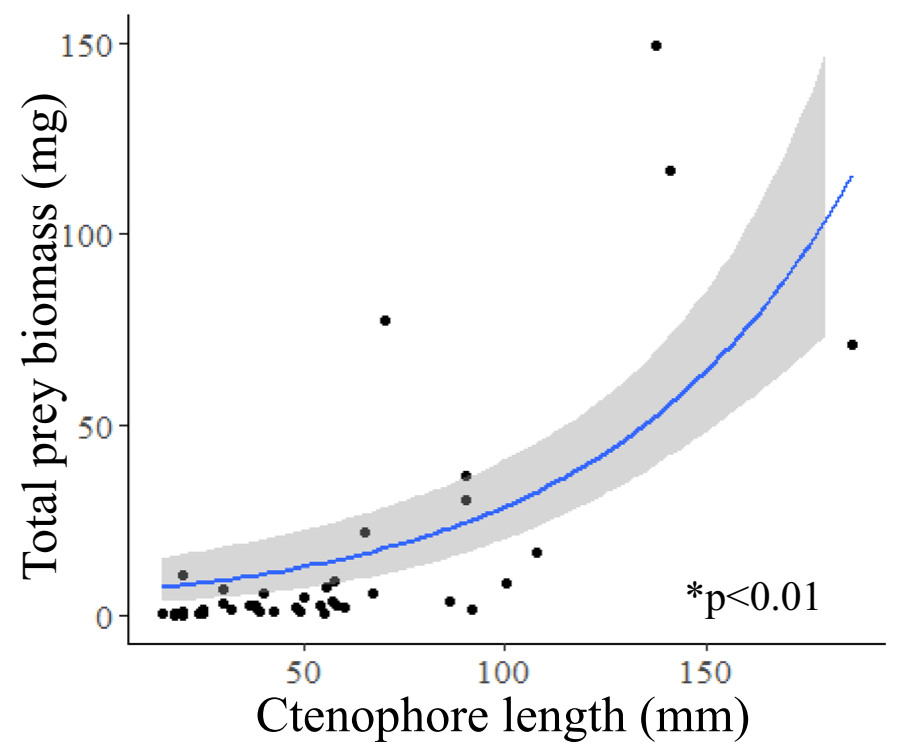
Logarithmic regressions showing a significant relationship between ctenophore length and total prey biomass in the gut (*p* < 0.01, r^2^ = 0.57). Grey shaded region shows 95% confidence interval. Two outliers were removed from the plot for visualization purposes only.

Ctenophore body length was compared to total prey biomass, average prey length, percent gut fullness, and number of prey per gut using linear regressions. The only significant relationship found was a positive logarithmic relationship between ctenophore body length and total prey biomass (logarithmic regression, *p* < 0.01, n = 44) (Fig. 8). Time of day and ctenophore size did not influence percent gut fullness (ANCOVA, F = 0.62, df = 1, 40, p = 0.43).

Plankton tows were taken at 0–1 m depth as that was the most feasible option given the time and resources available during dive trips. Data from day and night tows are shown in Table 2. The top three most abundant genera from the daytime tow were *Parvocalanus* (43.3%), *Paracalanus* (17.4%), and *Corycaeus* (11.2%). At night, *Parvocalanus* was again the most abundant species (48.1%), followed by *Oithona* and *Paracalanus* at 9.6% and 9.1%, respectively. Copepod species > 1 mm in length on average made up 28.6% of the copepod community in daytime samples and 9.1% of the copepod community at night.

**Table 2:** Estimates of number of each species found in situ and their proportions of the total copepod population. All species observed were found in both day and night samples.

Genus	Day		Night	
	ind m^−3^	Proportion of total (%)	ind m^−3^	Proportion of total (%)
*Acartia*	30	3.18	82	5.23
*Calanus*	38	4.03	79	5.05
*Pseudocalanus*	9	0.95	8	0.52
*Parvocalanus*	412	43.27	751	48.08
*Paracalanus*	166	17.39	142	9.06
*Labidocera*	6	0.64	12	0.78
*Temora*	6	0.64	19	1.22
*Oithona*	34	3.61	150	9.58
*Halicyclops*	12	1.27	8	0.52
*Oncaea*	51	5.30	93	5.92
*Corycaeus*	107	11.24	68	4.36
*Macro/Microsetella*	6	0.64	12	0.78
nauplii	75	7.85	139	8.89
**Total**	953		1563	

### Digestion time

**Figure 9:**
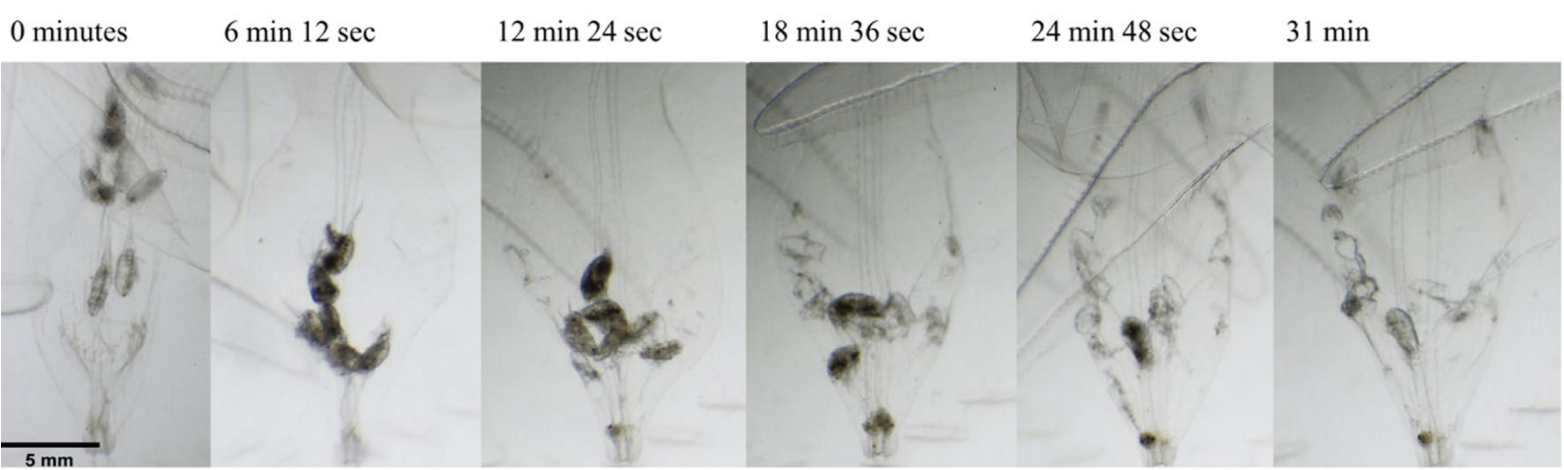
Sequential images displaying complete digestion to chitinous copepod skeletons in an *Ocyropsis* spp. individual. Scale bar in first image represents 5 mm.

*Ocyropsis* spp. individuals observed under a microscope showed that copepod prey moved to the bottom of the gut within the first 10 to 15 min and were then digested one or multiple at a time (Fig. 9). The average time for complete digestion of all prey in the gut at an ambient temperature of 25 °C was 44.19 min (S.D. 10.45) (n = 14). Ctenophore body length, which ranged from 9 to 25 mm, did not significantly affect digestion time (linear regression, *p* = 0.85, n = 14). The number of prey in a ctenophore gut also did not affect digestion time (linear regression, *p* = 0.26, n = 14).

### Predation rate and trophic impacts

*Ocyropsis* spp. predation rate was calculated using the formula from Pagès et al. (1996) which assumed a constant digestion time over 24 h. A high-end density (1 ind m^−3^) could potentially consume 629 copepods m^−3^ d^−1^. A low-end density (0.014 ind m^−3^) could consume 9 copepods m^−3^ d^−1^ (Table 3). An estimate of maximum predation effect was calculated using the high-end standing stock estimates from the plankton samples (nighttime data, Table 2), and a density of 1000 *Ocyropsis* spp. 1000 m^−3^ could potentially consume approximately 40.2% of the daily copepod standing stock while the low-end density of 14 *Ocyropsis* spp. 1000 m^−3^ could consume 0.56% of the standing stock per day (Table 3). Estimates of copepod carbon content from Kremer (1986b), average copepod biomass from gut content data, and predation rate were used to calculate the consumption of high-end densities of *Ocyropsis* spp. which could approximate 19.21 mg C d^−1^ while low-end densities could consume 0.27 mg C d^−1^ (Table 3).

**Table 3:** Predation impact estimates for high and low-end *Ocyropsis* spp. densities.

*Ocyropsis* spp. density (ind m^−3^)	Predation rate (copepods ingested m^−3^ d^−1^)	Predation effect (% of daily copepod standing stock consumed)	Carbon ingestion (mg C/d)
1	629	40.2	19.2
0.014	9	0.56	0.27

## Discussion

This study provides quantitative data for *Ocyropsis* spp. feeding mechanisms and in situ data for gut contents during both day and night to begin assessing their trophic role in oceanic waters. Previous studies qualitatively described the feeding pattern of *Ocyropsis* spp.^15^ whereby this animal uses a unique capture mechanism among lobate ctenophores: direct transfer from lobe to mouth and encounters involving the mouth actively grabbing copepod prey^24^. These previous observations are confirmed as *Ocyropsis* spp. is able to deploy its dexterous, prehensile mouth to effectively capture prey within the lobes (Figs. 2, 3) and quantitative assessments of predation are also provided. It should be noted that while *Ocyropsis* spp. are known to occasionally consume a wide variety of prey types and sizes^15^, this study focuses only on copepod prey because our field data showed recognizable prey in *Ocyropsis* spp. guts was almost exclusively copepods.

For example, mean speed of the mouth is less than 6 mm s^−1^ during predation events on copepods. Thus, while it may look rapid to the human eye, this is far below the escape swimming speeds exhibited by many copepods which are capable of moving at speeds of up to 500 mm s^−1^^25,26^. Our observations show that the mechanism of capture is thus not reliant on grabbing copepods from the water between the ctenophore lobes with the mouth, but rather aided by copepod contact with the ctenophore lobes. Copepods between the lobes swam only with a speed of 7.94 mm s^−1^ (S.D. 7.25), to which the average mouth speed (5.83 mm s^−1^ (S.D. 1.68)) is comparable (Table 1). This suggests that *Ocyropsis* is able to reduce copepod swimming activity either by trapping them against the lobes (lobes respond to contact by prey) and/or the use of some form of adhesion or chemical that acts to reduce copepod activity. This unusual form of predation using a prehensile mouth allows *Ocyropsis* to be highly effective predators without the use of prey capturing tentillae seen in other lobate species.

The presence of multiple prey has the potential to disrupt a raptorial type feeder such as *Ocyropsis* spp*.* more so than other lobates, since they lack tentillae, which would allow them to capture multiple prey simultaneously. Instead *Ocyropsis* spp*.* transfer one prey at a time directly from lobe to mouth^15,27^. So how is this ctenophore able to maintain such a high overall capture rate? The answer appears to be that *Ocyropsis* will modulate the number of attempts with the prehensile mouth depending on the number of prey present. For example, we did not observe any captures on the first attempt with the mouth with multiple prey, but the animals made up to 8 attempts at capturing the nearest copepod. This is in contrast to single copepod encounters in which ctenophores captured copepods on the first attempt 61% of the time and rarely made over 2 attempts, never exceeding 3 attempts (Figs. 3a, 5a, Table 1). This demonstrates *Ocyropsis* spp. can adjust its behavior to maintain high overall capture success when presented with multiple simultaneous prey. It is also interesting to note that the resulting increase in handling time due to making more attempts during multiple prey encounters is still lower than the handling time for most other lobates dealing with single prey^27,28^. It is not clear how often *Ocyropsis* spp*.* need to deal with multiple copepods simultaneously in nature, as oceanic waters contain characteristically low ctenophore prey densities compared to coastal zones^9,29^, however prey can be highly patchy and it appears that the unique prey capture mechanism of *Ocyropsis* spp*.* is still able to operate effectively in high density patches by increasing the number of attempts before aborting the attack which could serve as a means to maintain similar ingestion rates to single prey encounters.

Typically, the feeding sequence of a ctenophore involves capture of prey in sticky colloblast cells and retraction of tentillae and/or ciliary transport of prey to the mouth^15,27,30^. These feeding mechanisms result in a range of handling times ranging from 2.5 s for *Bolinopsis. infundibulum*^28^ to nearly 22 min for *Pleurobrachia bachei*^27^. Capture rates can also be quite high, with overall capture success rates up to 74% for *Mnemiopsis leidyi*^2,3^. We found *Ocyropsis* has a relatively fast mean handling time of 6.3 s when a single copepod was present between the lobes, but handling time increased by approximately 2.5-fold if multiple prey were present. Overall capture success rates were comparable to the highly effective coastal ctenophore, *M. leidyi*, with a 71% success rate with single prey present and 81% capture rates if multiple prey were present between the lobes. Thus, *Ocyropsis* spp. are able to capture prey with high efficiency despite the differences in feeding mechanics compared to coastal lobate ctenophores. Additionally, since encounter rates of planktivores are directly related to the time spent searching for prey and time spent handling prey^27^, the relatively short handling time of *Ocyropsis* spp. and their direct feeding mechanism may allow them to sample more water and encounter a larger proportion of the available prey population than other species.

### Diel patterns of prey consumption

Many planktivorous species exhibit higher gut fullness at night^31,32^, due to higher prey availability in surface waters as a result of a diel vertical migration^33,34^. In situ gut content images showed that *Ocyropsis* spp. had a significantly higher gut fullness at night (12.4%) compared to during the day (4.2%) (Fig. 7). *Ocyropsis* spp. also had higher numbers of prey per individual gut at night, although overall biomass was not significantly different between night and day (Fig. 7). This can be explained by differences in prey characteristics; prey observed in the gut during the day were significantly larger (Table 2). This may be due to an ability to feed more selectively during the day since overall prey densities are lower. It should also be considered that turbulence in surface waters is, on average, much lower at night compared to daytime^35^ and that even small amounts of turbulence can negatively impact ctenophore feeding^36,37^. Therefore, smaller prey may have a higher likelihood of evading detection of *Ocyropsis* during the day compared to night, especially since these animals are most frequently observed in the upper 15 m of oceanic waters.

Kremer, et al.^38^ estimates that *O. crystallina* requires 252 prey items to sustain itself. On average, *Ocyropsis* spp. in this study consume over 500 prey d^−1^. This exceeds their metabolic demands and suggests the observed population, on the western edge of the Gulf Stream, are likely to be actively growing and reproducing. The time required to digest prey items averaged 44 min for *Ocyropsis* which is faster than many, but not all, gelatinous zooplankton^39–41^. Digestion times of other gelatinous taxa span a range of times from 15 min to over 7 h at 20 °C^40^ and are impacted by size and number of prey per gut as well as temperature^39,42,43^. Digestion observations were performed at an ambient temperature of 25 °C and thus, these numbers represent a conservative estimate because the temperature of the water from which the animals were collected was 26.7–27.4 °C. *Ocyropsis* spp. would likely experience an increase in digestion rate with increased temperature.

Digestion time was not impacted by the number of prey in the gut or by ctenophore body length*.* This differs from trends seen in other gelatinous taxa, such as *A. aurita, M. leidyi,* and *B. infundibulum*, where increasing body size resulted in faster digestion time^39,40^ and where increasing number of prey in the gut leads to longer digestion times^39–41^. In this study however, ctenophores were offered only a few copepods to ingest, thus it is likely they were not fed enough prey to satiate and slow the digestion process. Also worth considering is that the metabolic rate of *O. crystallina* does not appear to be affected by body size^38^. Though metabolic rates were not measured, this aligns with our finding that body size had no significant effect on digestion time. Analysis of in situ gut contents showed a significant positive logarithmic relationship between ctenophore length and total prey biomass per gut (Fig. 8). Individuals smaller than 20 mm in this study typically had fewer than the average number of copepods per gut (19), and larger individuals were the main driver of this relationship. This suggests that small *Ocyropsis* (< 20 mm) cannot proportionally consume as much biomass as larger individuals and thus would not have as large of an impact on prey fields. Volume of gelatinous predators is known to directly affect encounter rates^27^, so as *Ocyropsis* spp. grow in length and volume, they encounter exponentially more water, and thus more prey, which allows larger individuals to consume proportionally more biomass.

Using data collected in this study and the estimated high end of naturally observed *Ocyropsis* spp. densities from the literature (1000 ind*.* per 1000 m^3^), this species could potentially consume 40.2% of the daily copepod standing stock, assuming continuous digestion time over 24 h (Table 3). However, at the lower end of observed natural densities (14 *Ocyropsis* spp. per 1000 m^3^), *Ocyropsis* spp. populations would consume less than 1% of the daily standing stock (Table 3). Alldredge^44^ estimated that all species of gelatinous zooplankton together typically consume less than 10%, but occasionally more than 50%, of prey standing stock each day. At high densities, *Ocyropsis* spp. alone appears capable of coming close to Alldredge’s high-end estimate, but the more commonly observed lower densities, fit best into Alldredge’s comprehensive range. Compared to *Ocyropsis* spp*.*, only coastal gelatinous taxa such as *M. leidyi*, *P. pileus*, and *C. quinquecirrha* are capable of consuming a higher proportion of the standing stock than this high end density estimate^45–47^. Thus, this study represents the first to demonstrate that an oceanic ctenophore, when at high natural densities, can have a strong trophic impact within open ocean ecosystems.

However, it is important to note that tropical and subtropical copepods have a complicated life history requiring days to weeks for full development. Thus, a predation rate of 40% daily could deplete oceanic copepod stocks in a short period of time. It is possible that some copepod species may accumulate at depths below where most *Ocyropsis* spp. are typically found (upper 15 m) which could provide a refuge from intense predation at the surface. It is also likely that lower densities of *Ocyropsis* spp. are more common than higher densities across large geographical areas, though we currently lack fine-scale spatial data of gelatinous plankton over large areas or through time to understand the scales of patchiness. The wide range in estimates for grazing of the daily standing copepod stock (0.56–40%) from low to high densities of these predators suggests that they do have the potential to impact open ocean environments in a strong manner, but how commonly this is occurring and over what spatial scale is still unknown.

Some gelatinous grazers such as salps produce dense fecal pellets that sink rapidly, exporting large amounts of carbon and nitrogen from surface waters^48^. Ctenophores, however, do not produce fecal pellets, so their waste is recycled in surface waters for further use by producers^49^. However, it is possible that biomass consumed by *Ocyropsis* makes it into the deep ocean through deposition to the seafloor in jelly falls^14,50^. By these means, ctenophores may play an important role in the global biological pump^50^. It is important to consider however, that though many individuals may be involved in jelly falls, their carbon content is relatively low (1.18%)^38^ because they are made of approximately 95% water^51,52^. On the other hand, since *Ocyropsis* spp. can be found at densities exceeding 1 individual per 1 m^3^, the amount of carbon exported from surface water may be significant in jelly fall events, especially considering the immense geographical scale this animal occupies on the planet^9^. Gelatinous zooplankton are additionally an important food source for fishes. Diaz Briz, et al.^53^ found that 39 of 107 oceanic fish species were consumers of gelatinous zooplankton, and members of suborder Stromateoidei were found to consume gelatinous zooplankton as their main nutrition source. Thus, *Ocyropsis* spp. may provide an important link between phytoplankton, zooplankton, and the rest of the epipelagic food web, as well as contribute to global oceanic cycling of carbon.

The ability to resolve details of the predator–prey interactions for an abundant oceanic ctenophore such as *Ocyropsis* spp.*,* will aid in a more complete understanding of the trophic interactions and impacts of this animal on oceanic planktonic ecosystems. The novel in situ methods utilized in this research allowed us to incorporate the ecological role of *Ocyropsis* spp. into the understanding of oceanic planktonic communities, and this work suggests that moderate to high abundances of *Ocyropsis* spp. are capable of exerting top-down control on copepod populations. Thus, *Ocyropsis* spp. may be acting to structure zooplankton communities in ways few other oceanic species can. This research provides an improved understanding of where this ctenophore fits into the epipelagic food web, but it is only one of many globally distributed oceanic ctenophore species and further research of other oceanic species using these methods is necessary to quantify and fully comprehend trophic ecology oceanic ctenophores.

## Data Availability

The raw data that were presented and analyzed for this manuscript are available at: 10.6084/m9.figshare.21801133.
